# Excess Mortality in Patients with Multiple Sclerosis Starts at 20 Years from Clinical Onset: Data from a Large-Scale French Observational Study

**DOI:** 10.1371/journal.pone.0132033

**Published:** 2015-07-06

**Authors:** Emmanuelle Leray, Sandra Vukusic, Marc Debouverie, Michel Clanet, Bruno Brochet, Jérôme de Sèze, Hélène Zéphir, Gilles Defer, Christine Lebrun-Frenay, Thibault Moreau, Pierre Clavelou, Jean Pelletier, Eric Berger, Philippe Cabre, Jean-Philippe Camdessanché, Shoshannah Kalson-Ray, Christian Confavreux, Gilles Edan

**Affiliations:** 1 Department of Epidemiology, EHESP Rennes, Sorbonne Paris Cité; CIC-P 1414, CHU Rennes, West Neuroscience Network of Excellence (WENNE), Rennes, France; 2 Department of Neurology, CHU Lyon, Observatoire Français de la Sclérose En Plaques (OFSEP), Lyon, France; 3 ReLSEP, Lorraine Register of MS, EA 4360, Department of Neurology, CHU Nancy, Nancy, France; 4 Department of Neurology, CHU Toulouse, Toulouse, France; 5 Department of Neurology, CHU Bordeaux, Bordeaux, France; 6 Department of Neurology, Hopitaux Universitaire de Strasbourg, Clinical investigation center (CIC INSERM 1434) and UMR INSERM 1119/Federation de Medecine translationnelle (FMTS), Strasbourg, France; 7 Université de Lille, Department of Neurology, Hôpital Roger Salengro, CHRU de Lille, Lille, France; 8 Department of Neurology, CHU Caen, Caen, France; 9 Department of Neurology, CHU Nice, Nice, France; 10 Department of Neurology, CHU Dijon, Dijon, France; 11 Department of Neurology, CHU Clermont-Ferrand, Clermont-Ferrand, France; 12 APHM, Hôpital de La Timone, Pôle de Neurosciences Cliniques, Department of Neurology, Marseille, France; 13 Department of Neurology, CHU Besançon, Besançon, France; 14 Department of Neurology, CHU de Martinique, Martinique, France; 15 Department of Neurology, CHU Saint-Etienne, Saint-Etienne, France; 16 Department of Epidemiology, EHESP Rennes, Sorbonne Paris Cité, Paris, France; 17 Department of Neurology, CHU Lyon, Lyon, France; 18 Department of Neurology, CHU Rennes, WEst Neuroscience Network of Excellence (WENNE), Rennes, France; University of Düsseldorf, GERMANY

## Abstract

**Background:**

Recent studies in multiple sclerosis (MS) showed longer survival times from clinical onset than older hospital-based series. However estimated median time ranges widely, from 24 to 45 years, which makes huge difference for patients as this neurological disease mainly starts around age 20 to 40. Precise and up-to-date reference data about mortality in MS are crucial for patients and neurologists, but unavailable yet in France.

**Objectives:**

Estimate survival in MS patients and compare mortality with that of the French general population.

**Methods:**

We conducted a multicenter observational study involving clinical longitudinal data from 30,413 eligible patients, linked to the national deaths register. Inclusion criteria were definite MS diagnosis and clinical onset prior to January, 1st 2009 in order to get a minimum of 1-year disease duration.

**Results:**

After removing between-center duplicates and applying inclusion criteria, the final population comprised 27,603 MS patients (F/M sex ratio 2.5, mean age at onset 33.0 years, 85.5% relapsing onset). During the follow-up period (mean 15.2 +/- 10.3 years), 1569 deaths (5.7%) were identified; half related to MS. Death rates were significantly higher in men, patients with later clinical onset, and in progressive MS. Overall excess mortality compared with the general population was moderate (Standardized Mortality Ratio 1.48, 95% confidence interval [1.41-1.55]), but increased considerably after 20 years of disease (2.20 [2.10-2.31]).

**Conclusions:**

This study revealed a moderate decrease in life expectancy in MS patients, and showed that the risk of dying is strongly correlated to disease duration and disability, highlighting the need for early actions that can slow disability progression.

## Introduction

A number of studies of mortality in multiple sclerosis (MS) have been conducted over the past 20 years, with more or less consistent results. Reported median survival times from MS clinical onset ranged from 24 to 45 years [1–16], with longer durations in more recent studies. Mortality in MS is of particular interest, as this neurological disease mainly affects young adults, most cases starting between age 20 and 40. All series have revealed excess mortality (1.3 to threefold) and reduced life expectancy (range: 6-14 years) in MS patients compared with the general population [1–11, 17, 18]. A lower age at onset is associated with a better prognosis, while results on sex remain controversial. The acknowledged increase in the female/male sex ratio (from 2:1 to nearly 3:1) is linked to the increased incidence among women [19–21], but may also raise the question of its potential relationship to differential survival between men and women, i.e. a better survival in women than in men.

In 2004, we carried out a study of 1879 French MS patients [5] that confirmed most results of the literature, although the standardized mortality ratio (SMR) was surprisingly low (1.3, 95% CI [1.0–1.7]). Several limitations may have accounted for this striking difference, including a short follow-up duration (13 years) and therefore a relatively youthful and low-disabled population at the end-of-study date. Indeed, in natural history studies, the median time to need walking aid is estimated to be around 20 to 25 years from MS clinical onset [22, 23]. There was also a potential selection bias, as patients were enrolled from a single MS referral center located in Rennes in Brittany region, Western France.

We therefore decided to initiate a large-scale multicenter study of mortality in MS in France, with the aim of measuring death rates and life expectancy in patients, describing causes of death, assessing prognostic factors for death, and comparing mortality in patients with the French general population.

## Methods

### Study population

Recruitment took place via the network of French users of EDMUS. Although EDMUS initially was a software dedicated to MS that was developed in France in the 1990s [24], it now corresponds to a large network of clinicians and researchers whose aim is to fight MS, by improving care and promoting research. In the software, demographic, and longitudinal clinical data are prospectively collected during each neurological appointment (some being retrospectively collected during the first appointment) and can be used for both medical follow-up and research purposes. A total of 15 MS centers agreed to participate in the present study, accounting for a potential of 33,000 MS patients at study launch (i.e. about one third of prevalent MS cases in France [25]). Inclusion criteria were defined as a definite diagnosis of MS according to either Poser [26] or McDonald [27, 28] depending on the period of diagnosis, and clinical onset of MS prior to January 1^st^ 2009 in order to have a minimum disease duration of 1 year at the end-of-study date, which was set as January 1^st^ 2010.

Each EDMUS database has been approved by the French data protection authority (‘Commission Nationale Informatique et Libertés’, CNIL). Furthermore, in accordance with French legislation, the present study was approved before its start by both the CNIL (Approval DR-2010-367) and the French advisory committee for data processing in health research (‘Comité Consultatif du Traitement de l’Information en matière de Recherche dans le domaine de la Santé, CCTIRS; Approval 10191). Those approvals were needed to link our dataset to the national registers containing death information. Then the data were analyzed anonymously.

### Death ascertainment and causes of death

As deaths are not exhaustively registered in the EDMUS databases, the clinical dataset was linked to two national registers. The first register was the national repertory for the identification of physical persons (‘Répertoire National d’Identification des Personnes Physiques’, RNIPP), where each individual born in France after 1890 is identified by sex, surname (maiden name for women), date and place of birth. This allowed us to obtain up-to-date vital status. We also obtained data from the national death register (‘INSERM-CepiDc’), which contains information provided in death certificates from 1968, including the date, place and causes of death, coded according to the International Classification of Diseases (World Health Organization). The underlying causes of death were categorized as follows according to ICD codes: MS (ICD-10 code G35), cancer, cardiovascular disease and stroke, infectious disease, respiratory disease, neurological disease except MS, suicide, digestive disease, accidental death, other disease, unspecified (e.g. cardiac arrest) or unknown (i.e. death certificate unavailable) causes.

### Prognostic factors for death

The primary endpoint was death, and the following parameters were considered as potential explanatory variables: age at MS clinical onset, sex, initial MS course, and period of MS onset. The initial MS course was defined as either relapsing onset or progressive onset [29]. The period of MS onset was categorized as < 1980, 1980-1989, 1990-1999, or 2000-2009.

### Comparison with the French general population

We calculated the individual probability of death for every patient during follow-up using life tables for the French general population, by sex and age for the period 1940–2009 (source: French National Institute for Demographic Studies). We calculated the number of expected deaths by summing the individual expected probabilities of death. In addition to the overall comparison between MS patients and the French general population, we also ran comparisons for patient subgroups (sex, age at MS clinical onset, initial MS course, follow-up duration, period of MS onset, and center) using standardized mortality ratios (SMRs). The individual expected probability of death was also used to draw the expected survival curve from MS onset. When comparing the expected curve with the observed one, the reduced survival time after MS clinical onset can be estimated approximately.

### Statistical analyses

Qualitative and quantitative variables are provided as numbers (percentage) and means ± standard deviation (*SD*), respectively. We calculated overall and subgroup mortality frequencies (number of deaths divided by number of patients; %) and mortality rates (number of deaths divided by number of person-years (PY) of follow-up per 1,000 PY). Sensitivity analysis was performed to estimate number of expected deaths in patients whose vital status was missing. Differences between subgroups were assessed using *t*, Anova or Fisher’s exact tests when appropriate. Kaplan-Meier estimates with log-rank tests were used to assess prognostic factors for the time between clinical MS onset and death, followed by a multivariate Cox proportional hazards model (backward method). As period of onset was closely linked to disease duration, it was not considered in the multivariate analysis. SMRs were equal to the number of observed deaths divided by the number of expected deaths. The 95% confidence limits of the SMRs were estimated using the Poisson distribution. All statistical tests were two-tailed, and statistical significance was set at the 5% level. Any missing data were excluded from the analyses. Statistical analyses were performed using STATA 11.

## Results

### Study population characteristics

**Fig 1:**
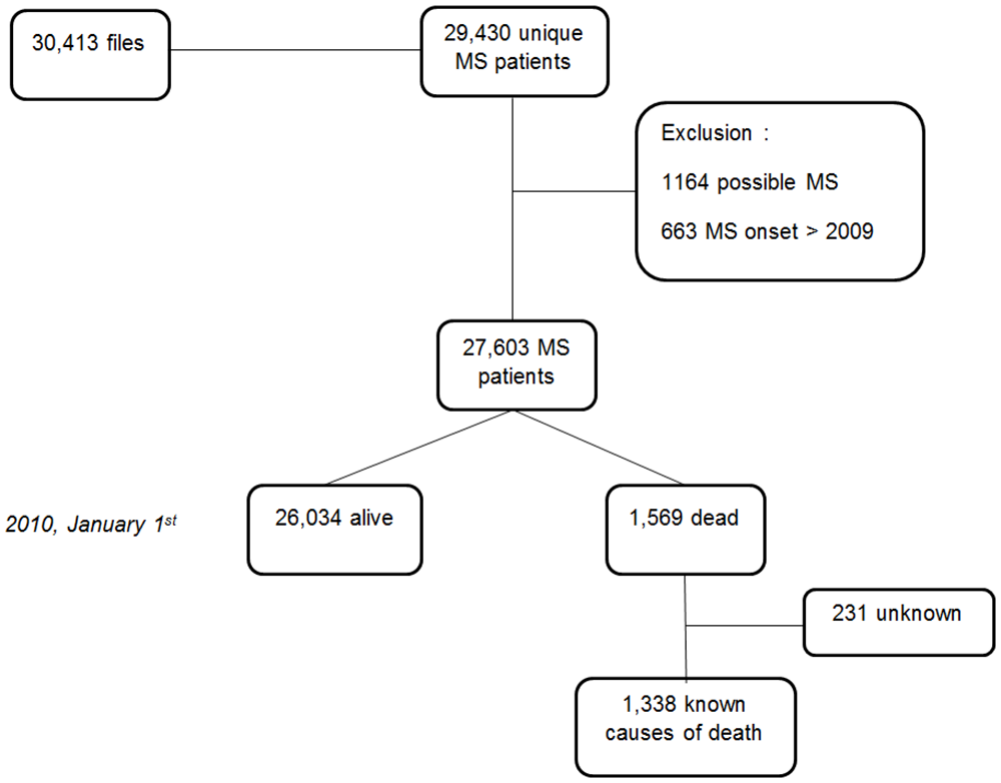
Flowchart of the study population. These patients had the classic characteristics of MS populations: F/M sex ratio 2.47 (19,656/7,947); mean age at MS clinical onset 32.8 ± 10.6 years; and 85.5% relapsing-onset MS (23,438/27,408; 195 missing values). As expected, mean age at onset was significantly higher and F/M sex ratio lower for progressive onset MS than for relapsing-onset MS (41.8 ± 10.8 vs. 31.2 ± 9.7 years, *t* test, *p* < 10^−4^, and 1.40 vs. 2.76, Fisher’s exact test, *p* < 10^−4^, respectively). MS = Multiple Sclerosis.

At study launch, 30,413 MS files were received from the 15 participating centers, equivalent to 29,430 MS patients after removal of between-centers duplicates. Then, 1,164 patients were excluded for “possible MS” diagnosis and a further 663 because MS onset occurred after 2009. The final study population size was 27,603 MS patients (Fig 1).

Mean follow-up duration was 15.2 ± 10.3 years from MS clinical onset, and the total number of patient-years of follow-up was 420,801. Mean age at last clinical information was 48.0 ± 10.3 years. Mean MS duration differed significantly according to age at MS onset (ANOVA *F* test, *p*<10^−4^): 19.9 ± 13.0, 15.8 ± 10.4, and 11.8 ± 7.7 years in the < 20, 20-40, and ≥ 40 years groups respectively. Furthermore, as expected, we found that the earlier the period of MS onset, the longer the disease duration (33.3 ± 9.7, 22.6 ± 5.1, 13.5 ± 4.0, and 5.6 ± 2.7 years for the < 1980, 1980-1989, 1990-1999, and 2000-2009 subgroups respectively, ANOVA *F* test, *p*<10^−4^). A total of 4,607 patients (16.7%) had unknown vital status at the end-of-study date because they were not found in the national repertory (‘RNIPP’). These patients were therefore included in the analyses as censored alive at the date of their last clinical information. Their mean follow-up duration was significantly lower than that of the patients with known vital status (12.2 ± 10.4 vs. 15.9 ± 10.2 years, *t* test, *p*<10^−4^). Their characteristics compared with those of died and alive patients are presented in Table 1.

**Table 1:** Comparison of demographic and clinical characteristics of patients with multiple sclerosis (MS) according to vital status at the end of the study.

	Vital status at the end of study		
Variable	Alive	Dead	Censored alive
	N = 21,427	N = 1,569	N = 4,607
Sex			
Male	6,152 (28.7%)	685 (43.7%)	1,110 (24.1%)
Female	15,275 (71.3%)	884 (56.3%)	3497 (75.9%)
Sex ratio F:M	2.5	1.3	3.2
Mean age at MS onset (± SD), years	32.4 ± 10.4	35.2 ± 11.8	35.9 ± 10.9
Mean age at last information (± SD), years	47.8 ± 12.5	56.4 ± 13.3	46.1 ± 13.1
Mean MS duration (± SD), years	15.5 ± 10.0	21.2 ± 11.7	12.2 ± 10.4
Initial MS course			
Relapsing	18,517 (86.7%)	1,137 (72.6%)	3,784 (84.4%)
Progressive	2,841 (13.3%)	430 (27.4%)	699 (15.6%)

### Mortality

During the follow-up period, a total of 1,569 deaths occurred (5.7%; 3.7 per 1,000 PY) in 884 women and 685 men, at a median age of 56 years (range: 19-96) and the median disease duration was 20 years (range: 1–88) (Table 2). Kaplan-Meier estimates revealed that 89.6% [88.9-90.2] and 72.3% [70.4-74.1] of MS patients were still alive 25 and 40 years after clinical onset respectively.

**Table 2:** Characteristics of the 1569 deaths categorized by underlying cause of death.

Cause of death	Number of deaths (/1569) (%)	F/M sex ratio	Median age in years at MS clinical onset (range)	Median age in years at death (range)	Median disease duration in years at death (range)	Number (%) of patients with relapsing onset
Total	1569 (100%)	884/685 1.29	34.3 (6-72)	56.0 (19-96)	19.7 (1-88)	1.137 (72.5%)
MS	700 (44.6%)	380/320 1.19	32.2 (10-72)	54.5 (22-88)	19.7 (1-63)	509 (72.8%)
Cancer	187 (11.8%)	120/67 1.79	38.0 (6-63)	58.8 (21-85)	19.2 (1-55)	152 (81.3%)
Cardiovascular disease / stroke	126 (8.1%)	60/66 0.91	38.1 (7-70)	61.0 (34-88)	22.2 (2-58)	89 (70.6%)
Infections	67 (3.0%)	41/26 1.58	33.3 (17-58)	63.9 (35-92)	24.6 (2-57)	39 (58.2%)
Suicide	47 (3.0%)	24/23 1.04	32.2 (14-54)	46.4 (19-69)	12.3 (2-45)	35 (74.5%)
Other causes^a^	211 (13.4%)	126/85 1.48	36.0 (11-72)	59.0 (20-96)	21.5 (1-59)	140 (66.3%)
Unknown^b^	231 (14.7%)	133/98 1.36	34.3 (11-72)	54.2 (19-88)	19.2 (1–53)	173 (74.9%)

^a^ Accidents, digestive diseases, respiratory diseases, neurological diseases other than MS, unspecified, and other causes.

^b^ Unavailable death certificates.

MS = multiple sclerosis; F = female; M = male.

### Causes of death

The underlying cause of death was known from the death certificates for 1338 dead patients, and were as follows: MS (n = 700; 52.3%), cancer (n = 187; 14.0%), cardiovascular disease or stroke (n = 126; 9.4%), infection (n = 67; 5.0%), suicide (n = 47; 3.5%), accident (n = 46; 3.4%), neurological disease other than MS (n = 34; 2.5%), digestive disease (n = 28; 2.1%), respiratory disease (n = 27; 2.0%), other cause (n = 40; 3.0%), and unspecified (n = 36; 2.7%). MS was mentioned as either underlying or contributive cause of death in 1017 death certificates (76.0%). Out of the 231 unavailable death certificates, 2 patients had died abroad and 11 certificates could not be found, while 218 patients were not found in the RNIPP registry (n = 4,607) even though their medical files indicated that they were dead.

Table 2 shows the patients’ characteristics according to their cause of death (less frequent categories collapsed into a broader “other causes” category). MS was the underlying cause of nearly half the deaths (700/1,569), and death occurred after a median MS duration of 20 years, at a median age of 56 years. Suicide was the category with the lowest age at death (median 46 years), shortest disease duration (median 12 years), and sex ratio closest to 1 (23 men and 24 women). In the cardiovascular disease or stroke category, the percentage of women was also quite similar to that of men (F/M sex ratio 0.91), whereas it was considerably higher for cancer-related deaths (F/M sex ratio: 1.79). In all, 67 cancer-related deaths occurred in men (most frequent: 23 lung, 6 colorectal), and 120 in women (most frequent: 28 breast, 21 lung, and 14 colorectal).

### Prognostic factors

Univariate assessment of the prognostic factors for death revealed that the risk of death was higher among men, patients with later clinical onset, those with primary progressive MS, and those with MS onset in an earlier period. These results were confirmed using a Cox multivariate analysis (Table 3).

**Table 3:** Mortality rates and survival probabilities 25 years after MS clinical onset.

Subgroups	Number of deaths (%)	Death rate (per 1000 patient-years) [95% CI]	Probability of being alive 25 years after MS clinical onset (%) [95% CI]	Adjusted odds ratio [95% CI]	p-value (Logrank test)
Overall	1,569 (5.7%)	3.73 [3.55–3.92]	89.6 [88.9–90.2]	n.a.	n.a.
Sex					10^−4^
Male	685 (8.6%)	5.53 [5.13–5.96]	84.9 [83.4–86.2]	1.67 [1.51–1.85]	
Female	884 (4.5%)	2.98 [2.79–3.18]	91.7 [91.0–92.4]	1	
Age at MS onset (years)					10^−4^
<20	134 (4.9%	2.45 [2.07–2.90]	95.1 [93.8–96.2]	1	
20–40	925 (5.1%)	3.23 [3.03–3.45]	91.4 [90.6–92.1]	1.81 [1.50–2.18]	
≥40	510 (7.5%)	6.38 [5.85–6.96]	76.1 [73.3–78.6]	4.84 [3.94–5.94]	
Initial course					10^−4^
Relapsing	1,137 (4.9%)	3.16 [2.98–3.34]	91.6 [90.9–92.2]	1	
Progressive	430 (10.8%)	7.33 [6.67–8.06]	73.3 [74.7–79.6]	1.73 [1.53–1.95]	
Year of onset				Not included	10^−4^
<1980	327 (18.2%)	5.44 [5.04–5.89]	93.7 [92.8–94.5]		
1980–1989	485 (9.1%)	4.05 [3.70–4.42]	88.2 [87.0–89.3]		
1990–1999	380 (3.7%)	2.78 [2.51–3.07]	n. a.		
2000–2009	73 (0.8%)	1.52 [1.21–1.91]	n. a.		
MS duration (years)			n. a.	Not included	10^−4^
0–10	257 (2.6%)	1.11 [0.98–1.26]			
10–20	543 (5.4%)	4.46 [4.10–4.85]			
20–30	447 (8.9%)	9.27 [8.45–10.17]			
30–40	216 (11.0%)	14.11 [12.35–16.12]			
>40	106 (15.1%)	26.48 [21.89–32.03]			

MS = multiple sclerosis; CI = confidence interval; n.a. = not applicable

### Comparison with the French general population

**Fig 2:**
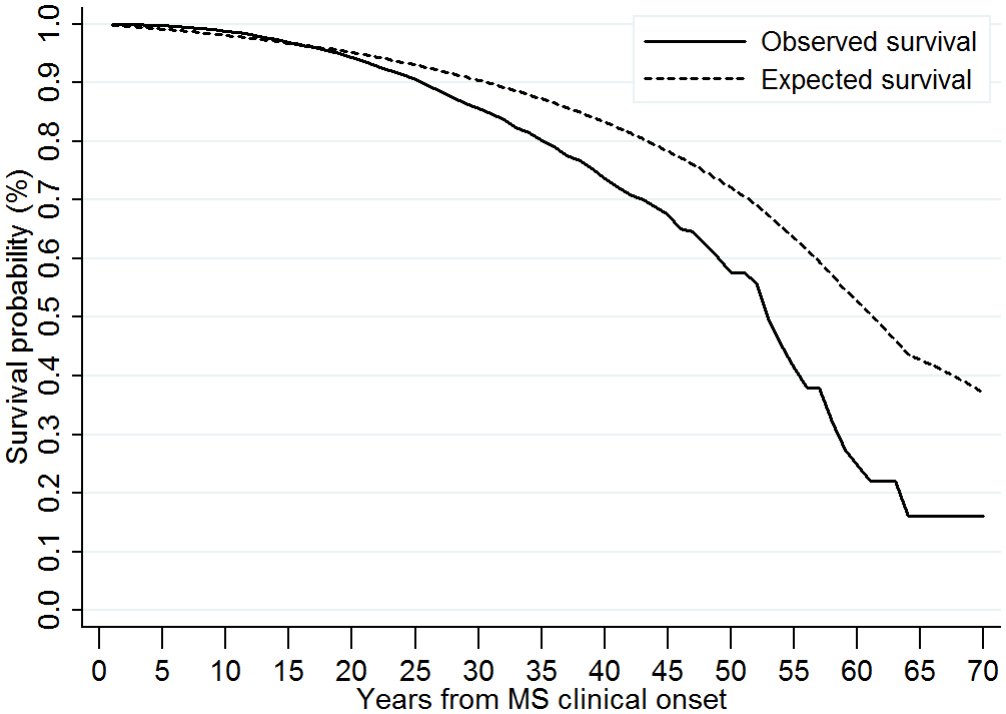
Comparison of the survival in MS patients with the survival of the French general population. Kaplan-Meier survival estimates according to time from MS clinical onset (years). MS = Multiple Sclerosis.

The overall SMR was 1.48 (95% CI [1.41-1.55]), resulting in 48% excess mortality in MS patients compared with the French general population. The survival curves (Fig 2) show that survival in MS patients was similar to that of the general population for the first 20 years of the disease. The two curves then begin to diverge, and the resulting gap can be estimated at around 7 years. Indeed, the observed median survival time was estimated to be around 53–54 years from MS onset, while the expected one in the French general population would have been about 61 years. Similarly, respectively 60% and 70% of patients were still alive after 49 and 43–44 years of MS duration, while these figures would have been observed in the French general population after 56–57 and 51 years.

Different SMRs were observed across the 15 participating centers. In Rennes, the SMR was 1.99 [1.77-2.25]. In Lyon, which is the original EDMUS center and largest referral center for MS in France, the SMR was 2.15 [1.98-2.34], and up to 2.78 [2.52–3.06] if considering historical cohort only [22]. In Lorraine region, the SMR was surprisingly low, at 0.88 [0.73-1.05]—the only one below 1 (but non significantly).

As shown in Table 4, excess mortality was higher in women than in men, and in patients with earlier rather than later MS clinical onset, while death rates were lower in these subgroups. On the opposite, patients with progressive onset had both higher mortality rates and higher excess mortality than patients with relapsing onset.

**Table 4:** Overall and subgroup comparisons of mortality between MS patients and the French general population using Standardized Mortality Ratios (SMR estimates with 95% CI, Poisson distribution).

	Number of patients	Observed deaths	Expected deaths	SMR [95% CI]
Overall	27,603	1,569	1060.64	1.48 [1.41–1.55]
Sex				
Male	7,947	674	524.67	1.30 [1.21–1.40]
Female	19,656	885	535.97	1.65 [1.55–1.76]
Age at MS onset (years)				
<20	2,746	134	62.19	2.15 [1.81–2.54]
20–40	18,089	925	581.39	1.59 [1.49–1.70]
≥ 40	6,768	510	417.06	1.22 [1.12–1.33]
Initial MS course				
Relapsing	22,438	1,137	797.23	1.43 [1.35–1.51]
Progressive	3,970	430	256.91	1.67 [1.52–1.84]
MS onset ≤ 2000	19,076	1,498	986.87	1.52 [1.44–1.60]
MS onset ≥ 1975	25,571	1,142	769.44	1.48 [1.40–1.57]
1^st^ appointment^a^ ≥ 1992	19,540	679	596.40	1.14 [1.06–1.23]
MS duration ≥ 20 years	8,475	1,569	711.96	2.20 [2.10–2.31]
MS duration ≥ 10 years	17,874	1,569	973.04	1.61 [1.53–1.69]
Patients censored alive excluded	22,996	1,569	917.97	1.71 [1.63–1.79]

^a^ 1^st^ appointment = 1^st^ appointment with a neurologist;

Abbreviations: CI = confidence interval; MS = multiple sclerosis; SMR = standardized mortality ratio.

If considering the overall death rate (1,569/364,700 = 4.3 per 1000 PY) in the subgroup of patients without updated vital status (*n* = 4,607), a total of 241 deaths may be added, leading to an overall number of 1,810 deaths and an overall SMR of 1.71 [1.63–1.79].

## Discussion

To our knowledge, SURVIMUS is one of the largest studies of mortality in MS that have been conducted so far, as more than 27,000 MS patients were included, representing about one third of French prevalent cases.

In the present study, deaths were captured by linking the dataset to two national registers. When the study started, only 500 deaths were notified in the EDMUS databases. This highlights the need for linkage to official national death registers in this kind of epidemiological study, in order to avoid underestimating the event of interest. Even so, a number of patients (*n* = 4,607) could not be traced in the French national register, either owing to a missing birthplace or because they were born outside France, or there were suspected mistakes in the maiden name (consistent with the high proportion of women). As this subgroup was older than the group with known vital status, it may lead to under-estimation of mortality, but without changing the key finding of our study. Indeed, if assuming they have the same mortality risk as the 22,996 patients who were identified in the register, the excess mortality compared with the French general population increased from 16% (from 1.48 to 1.71).

Our results confirm that MS patients are characterized by excess mortality, compared with the general population, but to a lesser extent (SMR 1.48; sensitivity analysis 1.71) than reported in previous studies (SMR up to 3) [2–4, 9, 10, 17]. We showed that life expectancy in patients is probably reduced by 7 years. More especially, we were able to distinguish between two phases in the risk of dying in MS patients: from MS clinical onset to 20 years, the risk is not higher in MS patients than in the French general population, whereas from 20 years of disease duration onwards, excess mortality does exist, and can probably be linked to higher disability. This reduction in life expectancy is one of the lowest estimates in the literature, and similar to North American studies [4, 6, 18], and the observed 25-year survival probability is one of the highest estimates. This may reveal an improvement in the survival of MS patients over time, as previously suggested [8]. However, we cannot assess how far, if at all, factors such as disease-modifying drugs (DMDs) or multidisciplinary MS management account for this improvement. Indeed, a long-term follow-up of a clinical trial has found an increased survival in early treatment group compared to delayed treatment group [30]. DMDs were not assessed in our study because this complex topic requires a specific design which takes into account lack of randomization in real-life study. However, we should also remember that a proportion of 89% of patients alive at 25 years of disease duration was already found in a Turkish study [13] published in 1998, i.e. before the treatment era.

We found that excess mortality was strongly correlated with disease duration, as the longer the disease duration, the greater the probability of death. We can assume that the overall SMR was lower than that of other series owing to the shorter disease duration of our MS population (mean 15 years). This is consistent with results of the first French EDMUS-user centers. In Rennes, the SMR was 1.99, that is much higher than the SMR (1.3) we had observed in our previous study [5], but the mean follow-up duration was also far longer (18 vs. 13 years). Similar results were observed in Lyon (whole and historical cohort). The low value of SMR in Lorraine region may be attributed to the recent nature of this MS population-based registry [31], meaning that although they are now close to achieving the exhaustive registration of prevalent cases, some patients have died before having the opportunity to be included in the database. By the way, the risk of dying for a patient before enrollment in the EDMUS database may apply in all the participating centers and can contribute to the lower excess mortality observed in the present study compared with other series. We can wonder if disease duration might be considered as a proxy of disability level (not available here), as disability was found as a prognostic factor for death [32] and low disability level was shown to be associated with a low SMR [5, 6]. But heterogeneity in the MS course is well-known, and many patients will live long lives without life-threatening disability.

Finally, a selection bias in patients’ enrollment towards a less malign course or an earlier diagnosis cannot be excluded too as the participating centers are considered as MS expert centers, as well as an immortal time bias (patients need to survive a sufficient amount of time to be enrolled in the database) as discussed above.

We observed that excess mortality was higher in patients who were young at MS onset and in women, even though these two subgroups had lower mortality rates. The fact that observed mortality may be low and excess mortality high suggests that excess mortality may be due to MS and not attributable to other factors such as demographics. Regarding the 1,338 known causes of death, MS was the underlying cause of death notified in 52.3% of deaths certificates, followed by cancer (14.0%) and cardiovascular disease (9.4%), mostly consistent with the literature. We can assume that the MS category includes complications of MS such as sepsis, aspiration pneumonia, and other events that are seen in individuals with severe disability. If pneumonia (ICD-10 code J690), tetraplegia (G825), and paraplegia (G822) as underlying cause were considered as MS, 18 cases would be added, leading to a proportion of deaths due to MS of 53.7%. Cardiovascular deaths were not so frequent, in accordance with the low mean age of the population at last information. The frequency of suicide (3.5%) was lower than in other series [2, 9, 17, 33], but some of them have also included accidents in this category. We can surmise that some of the deaths classified as accidental deaths or unspecified deaths were suicides. When we included some potential hidden suicides, we obtained a total number of 79, leading to a frequency of 5.9%. In the most recent studies, suicide was considered to be MS-related and included in the MS cause category [8]. Had we done so, MS would have accounted for 55.8% to 58.2% of deaths.

Next steps will consist of a further analysis of causes of death, not just underlying, but also contributory, and their combination. Moreover, attempts of defining “MS-related” death are ongoing. Cause-specific mortality rates of the French general population will be used and should help us to clarify the relationship between specific causes of death and excess mortality. Moreover, we are currently collecting additional data for suicide cases, in order to look at socioeconomic characteristics (employment, marital status, etc.), mental health status, and trends over time.

To conclude, the results of the SURVIMUS study show that there is a moderate decrease in life expectancy for MS patients, and that the risk of dying is strongly correlated with disease duration, and probably disability progression, highlighting the need for actions that can delay disability progression. Even though excess mortality starts only after 20 years of disease duration, interventions such as DMTs or multidisciplinary care should be started as soon as possible in the disease course in order to maximize their efficacy in slowing down disability progression [34]. We now have access to precise and up-to-date reference data about survival in MS in France, which is crucial for neurologists, patients, and their families.
